# SCEAF-UNet: Medical image segmentation based on spatial-channel feature enhancement and adaptive fusion

**DOI:** 10.1371/journal.pone.0345538

**Published:** 2026-03-25

**Authors:** Lingyun Zhao, Yanping Chen, Chao Wang, Yang Yu

**Affiliations:** 1 School of Science, Shandong Jianzhu University, Jinan, Shandong, China; 2 Department of Inspur Electronic Information Industry Co., Ltd., Jinan, Shandong, China; 3 Department of PET/CT, Shandong Cancer Hospital and Institute, Shandong First Medical University and Shandong Academy of Medical Sciences, Jinan, Shandong, China; Samsun University: Samsun Universitesi, TÜRKIYE

## Abstract

Achieving a balance between spatial and channel feature representations is critical for improving performance in medical image segmentation. This paper proposes the spatial-channel feature enhancement and adaptive fusion (SCEAF) module. This module is composed of a multi-scale spatial attention gated block (MSAGBlock) and a channel attention modulation block (CAMBlock) operating in parallel. The MSAGBlock enhances spatial detail recovery, while the CAMBlock strengthens channel feature discrimination, and achieves dynamic fusion between the two blocks by means of gated weighting. Building upon the RWKV-UNet backbone network, we integrate the SCEAF module into the decoder to construct the novel SCEAF-UNet architecture. In addition, we introduce the lightweight edge attention fusion (EAF) module at the skip connection, which captures edge information and highlights structural contours, helping the network better delineate organ borders. Experiments conducted on the public Synapse and ACDC datasets indicate that SCEAF-UNet significantly surpasses current models of various architectures. Further ablation experiments verify the effectiveness and scalability of the designed modules, which are suitable for integration into diverse medical image segmentation architectures.

## 1. Introduction

As a fundamental technology in intelligent healthcare, medical image segmentation contributes considerably to clinical diagnosis [1,2], pathological analysis [3], and computer-assisted surgery [4,5]. This technique has evolved from traditional threshold segmentation [6] to deep learning [7] end-to-end models, undergoing continuous development and refinement. At this stage, it has become relatively mature in clinical applications, effectively improving physicians’ diagnostic efficiency and accuracy.

Classical end-to-end convolutional neural network (CNN) models [8–13] employ an encoder-decoder structure and utilize skip connections to fuse features from different layers, achieving notable success in medical image segmentation tasks. Benefiting from their hierarchical convolutional design, these CNN models can effectively capture rich semantic information. Nevertheless, the intrinsic locality of convolution restricts feature interaction to limited receptive fields, making it difficult to model long-range dependencies explicitly [14]. This constraint becomes evident when handling complex anatomical structures, low-contrast regions, or large-scale lesions, where both boundary delineation and global semantic understanding remain suboptimal.

To overcome the limitations of insufficient global context modeling, multiple studies [15–19] have integrated self-attention mechanism into segmentation networks. By establishing dependencies between arbitrary spatial locations within feature maps, this mechanism enables a more comprehensive understanding of global semantics and demonstrates clear advantages in handling complex segmentation tasks. However, the incorporation of the self-attention mechanism substantially increases model parameters and computational costs [20], limiting its applicability in real-time clinical settings.

In recent years, researchers have introduced lightweight attention mechanisms to attain a better trade-off between modeling capacity and computational overhead. From the perspective of the feature dimensions they focus on, the attention mechanisms can be divided into channel attention (CA) [21] and spatial attention (SA) [22]. CA mainly models the correlation between different features in the channel dimension, highlights the responses of task-relevant channels through adaptive weighting, and suppresses the redundant information at the same time [23]. SA emphasizes the salient regions in the spatial dimension, enhancing key spatial location information and suppressing background interference by generating pixel-level or region-level weight maps [22]. Even so, existing attention methods [23–25] still exhibit notable limitations. For instance, relying solely on CA provides limited perceptual capability to spatial information, making it challenging to accurately characterize the local spatial structure of organs or lesions [23,24]. Conversely, using only SA cannot fully exploit the semantic features between channels, which may compromise the expression of overall structural information [25]. For this reason, some methods [22,26,27] attempt to achieve synergistic optimization of spatial and channel feature dependencies by jointly modeling both. These methods still have the following two shortcomings: on the one hand, their spatial and channel attention mechanisms are usually combined in a simple addition or cascade manner, which cannot achieve deep fusion of spatial and channel features [22,27]; on the other hand, these methods focus on strengthening the global spatial information and channel modeling, but they are often insufficient to portray local spatial structures and small-scale targets [26].

To resolve the issues mentioned above, this paper proposes the spatial-channel feature enhancement and adaptive fusion (SCEAF) module. Specifically, we design two functionally complementary submodules: the multi-scale spatial attention gated block (MSAGBlock) and the channel attention modulation block (CAMBlock). The MSAGBlock applies multi-scale depthwise separable convolutions on both global and local inputs to capture spatial information at different scales, and finely modulates local spatial features under the guidance of global information through parallel Sigmoid and Tanh gates. The CAMBlock mainly combines a channel MLP with a squeeze-and-excitation (SE) module [23], strengthening its capability to express and filter channel features. We fuse the outputs of MSAGBlock and CAMBlock through gated weighted summation to form the complete SCEAF module, achieving the optimization and balance of spatial and channel features, as well as global and local details. Additionally, we introduce the edge attention fusion (EAF) module to explicitly enhance edge feature representations. We construct the SCEAF-UNet model, primarily utilizing the SCEAF modules and supplementing it with the EAF modules, effectively boosting segmentation performance while maintaining computational efficiency. In conclusion, this study makes the following key contributions:

1. Proposing the SCEAF module, which fuses MSAGBlock and CAMBlock to achieve joint modeling of spatial details and channel semantics.
2. Introducing the EAF module to improve edge feature expression capabilities and promote fine segmentation.
3. Constructing an efficient segmentation network, SCEAF-UNet, that balances computational efficiency and segmentation precision.
4. Experimental findings on two commonly adopted medical datasets suggest that SCEAF-UNet outperforms former state-of-the-art (SOTA) methods, confirming its utility and competitiveness.

## 2. Related work

### 2.1. Architecture for medical image segmentation

**UNet-based architectures:** Since the birth of the U-Net [8], its concise and efficient structural design has inspired a wide range of subsequent research and spawned several variants. Weighted Res-UNet [10] integrates residual connections with a weighted attention mechanism to improve the discriminative ability of features in key regions by adaptively assigning weights to different feature layers. Attention-UNet [28] incorporates an attention gate mechanism that dynamically weights features to selectively enhance lesion regions. Dense-UNet [11] employs a dense block to boost feature reuse and gradient flow, thereby optimizing the feature representations. U-Net++ [12] mitigates semantic discrepancies by implementing nested skip connections between encoder-decoder paths at different depths. U-Net3+ [13] further improves on U-Net++ to achieve more efficient multi-scale semantic fusion with better segmentation accuracy through full-scale feature aggregation and deep supervised learning. Although these improved methods enhance the ability of feature extraction or multi-scale feature fusion, they are still centered on convolution and lack the capability to model the global context.

**Transformer**-**based UNet architectures:** In 2020, Chen et al. [15] initiated the incorporation of Transformers [29] into U-Net. Their proposed hybrid architecture, TransUNet, serializes features extracted by CNNs before feeding them into Transformers to model global dependencies while preserving local features. This approach opened new avenues for research on Transformer-based variants of U-Net. Subsequently, Cao et al. [16] introduced Swin Transformer with its sliding-window mechanism as the core of the encoder-decoder, and proposed Swin-Unet. This method achieves efficient modeling of both local and global features while facilitating cross-window information interaction. Xu et al. [17] proposed LeViT-Unet, whose encoder is constructed based on LeViT blocks. It extracts local texture and global semantic information by combining the advantages of CNNs and Transformers, while effectively reducing computational overhead. Wang et al. [18] proposed MT-UNet by embedding a mixed Transformer module at different stages of the encoder. This module first computes attention within local windows to capture local context, then calculates global attention to model global semantics, and balances dependencies between tokens of different distances through a Gaussian weighting mechanism, enabling the model to exhibit certain advantages in multi-scale feature modeling. Yan et al. [19] further proposed AFTer-UNet, which simultaneously models intra-layer and inter-layer dependencies through axial fusion of Transformers, thereby boosting the model’s global understanding of three-dimensional anatomical structures. Despite the advances achieved by these methods in integrating local and global information, they remain limited in modeling dependencies along the channel dimension and have room for further improvement in capturing fine spatial details.

### 2.2. Improvements based on attention mechanisms

Attention mechanisms are highly favored in the field of computer vision due to their powerful capabilities in feature selection and information weighting [30]. Woo et al. [22] developed the convolutional block attention module (CBAM), which enhances the representational power of key channels and spatial locations by combining CA and SA for weighting feature maps. Despite its effectiveness, CBAM connects CA and SA using a simple sequential cascade, which restricts the interaction between spatial and channel features. DAU-Net [31] promotes spatial relationships within local features by integrating CBAM with a position attention module. However, these two modules are processed through parallel addition rather than deep fusion, resulting in some feature redundancy. SA-UMamba [25] proposed a residual spatial state-space block that combines the Mamba module with a receptive field attention convolution module, aiming to model global dependencies while capturing spatially diverse local features. Nevertheless, this method mainly focuses on spatial information modeling and is weak in portraying semantic features of channels. Hou et al. [32] proposed the coordinate attention module, which splits the global average pooling into horizontal and vertical encodings by introducing coordinate information into the attention mechanism, thus preserving the position information when capturing long-range dependencies. Even so, this method lacks adequate perception of features at different scales and has limitations in expressing local details.

Therefore, the focus of this paper is to make full use of the advantages of the attention mechanism and develop an efficient attention method that compensates for the above-mentioned shortcomings.

## 3. Approach

**Fig 1:**
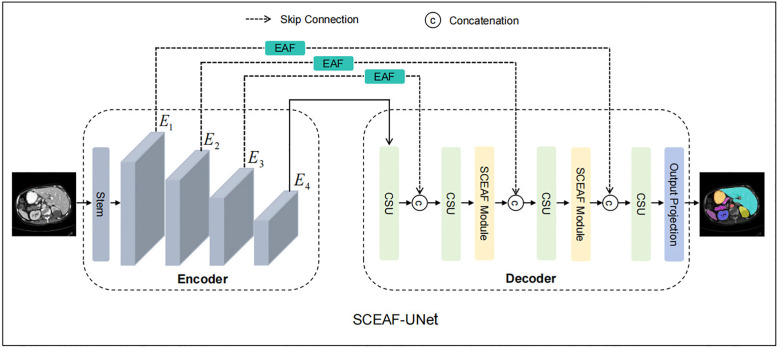
Overall architecture of SCEAF-UNet.

As shown in Fig 1, the proposed SCEAF-UNet architecture inherits the classic encoder-decoder design, which efficiently supports segmentation tasks of complex structures in medical images. The encoder section on the left extracts multi-scale features layer by layer to capture rich semantic information. The skip connection section in the middle introduces the EAF modules to refine the boundaries of encoded features before passing them to the decoder. The decoder section on the right restores spatial resolution using the channel shuffle upsampling block (CSUBlock). Following the second and third stages of the CSUBlock, an SCEAF module is applied to further optimize the spatial and channel representations of decoded features, enabling precise feature reconstruction and efficient fusion.

### 3.1. Encoder

In this study, the encoder is based on the RWKV-UNet Enc-B architecture [33]. Unlike standard Transformers [29], this design integrates the receptance weighted key value mechanism [34] with linear computational complexity, which allows efficient modeling of global dependencies while substantially reducing computational overhead. The Stem section of the encoder performs preliminary feature transformation, generating the feature representations required for subsequent stages. During the experiments, we acquire features from the four stages of the backbone network, labeled as E_1_, E_2_, E_3_, and E_4_.

### 3.2. Skip connection

**Fig 2:**
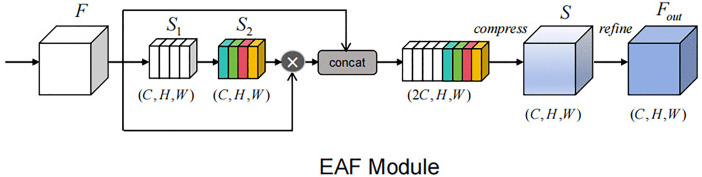
Edge attention fusion module. Different colors represent different weights assigned to each channel.

In conventional CNN models, multi-scale features extracted at the encoding stage are directly fused with the corresponding decoder features through skip connections, which contain a significant amount of redundancy and background noise, impairing the precise reconstruction of target boundaries [35]. To mitigate this issue, we propose the EAF module, as shown in Fig 2. This module leverages edge convolutions to extract boundary information and employs an edge attention mechanism to refine encoded features, enabling the network to concentrate on the edges of important structures. At the same time, the EAF module reduces redundant information by adaptively merging edge-enhanced features with the original feature maps. The specific operational process is as follows:

First, we use a 3 × 3 depthwise convolution (DWConv_3 × 3_(·)) on the input encoded features F ∈ R^C×H×W^ (where C denotes the number of channels; H and W represent spatial dimensions, as defined below), simulating a high-pass filter to extract edge responses:


$$\begin{array}{c} S_{\text{1}}\;=\;{DWConv}_{\text{3}\times \text{3}}(F) \end{array}$$ (1)

After extracting the edge features S_1_, an edge attention mechanism is introduced. Edge attention weights S_2_ are generated by two layers of 1 × 1 convolutions, a GELU and a Sigmoid activation function. The first 1 × 1 convolution layer performs channel compression mapping, denoted as Conv_1_: R^C^ → R^C/r^; the second 1 × 1 convolution layer conducts channel restoration mapping, denoted as Conv_2_: R^C/r^ → R^C^ (r represents reduction ratio).


$$\begin{array}{c} S_{\text{2}}\;=\;Sigmoid(\overset{\text{1}\times \text{1}}{\underset{\text{2}}{Conv}}(GELU(\overset{\text{1}\times \text{1}}{\underset{\text{1}}{Conv}}(S_{\text{1}})))) \end{array}$$ (2)

Next, the original encoder features are concatenated with the weighted features (concat(‧)). The concatenated features are then passed through a 1 × 1 convolution to compress the number of channels from 2C back to C, followed by batch normalization (BN) and GELU activation, resulting in the fused feature S:


$$\begin{array}{c} S\;=\;GELU(BN({Conv}_{\text{1}\times \text{1}}((concat(F,\;F\;⨀\;S_{\text{2}}))))) \end{array}$$ (3)

Here, ⊙ denotes element-wise multiplication (as defined below).

Finally, we perform edge refinement on the fusion result S using a 3 × 3 depthwise convolution (DWConv_3 × 3_(‧)), BN and a GELU activation function, outputting the feature F_out_:


$$\begin{array}{c} F_{out}\;=\;GELU(BN({DWConv}_{\text{3}\times \text{3}}(S))) \end{array}$$ (4)

### 3.3. Decoder

**Fig 3:**
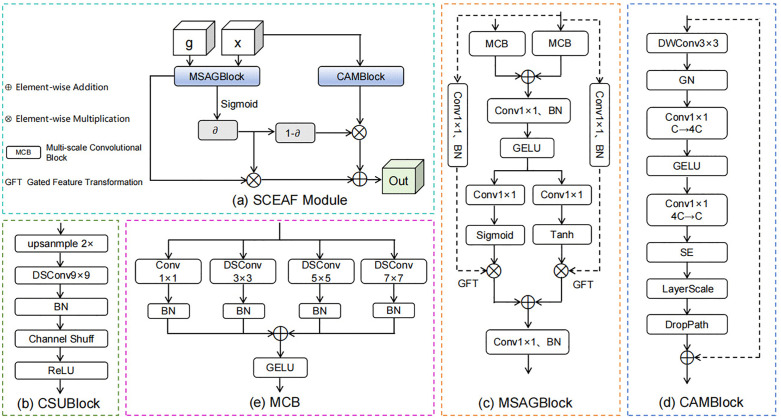
Network structure of the modules in the decoder. (a) Spatial-channel feature enhancement and adaptive fusion (SCEAF) module, (b) channel shuffle upsampling block (CSUBlock), (c) multi-scale spatial attention gated block (MSAGBlock), (d) channel attention modulation block (CAMBlock), (e) multi-scale convolution block (MCB).

This paper constructs a decoder comprising CSUBlocks and SCEAF modules, as shown in Fig 3(a) and 3(b).

The CSUBlock first applies bilinear interpolation upsampling (up(∙; 2)) on the input features d ∈ R^C×H×W^ to restore spatial resolution. Subsequently, a 9 × 9 depthwise separable convolution (DSConv_9 × 9_(‧)) achieves spatial feature extraction and channel information mixing, combined with BN for stable training. To enhance cross-channel information interaction, we introduce a channel shuffle operation (CS(‧)) [36] within the CSUBlock, effectively mitigating the channel independence introduced by grouped convolutions. Finally, after ReLU activation, the output yields more discriminative features D_CSU_ ∈ R^C′×2H×2W^.

The SCEAF module is embedded within the second and third stages of the decoder to optimize the preliminary features of the current decoding layer. This module consists of two branches. One branch feeds the global guiding feature g (from the encoder) and the local feature x (from the decoder) into the MSAGBlock. Both input features are initially passed through the multi-scale convolution block (MCB), and then processed by other components. Finally, this branch produces the output Y_MSAG_(g,x). The other branch enters the CAMBlock, where the input is solely the local feature x, producing the output Y_CAM_(x). Here, Y_MSAG_(g,x) undergoes Sigmoid activation to generate element-wise weight coefficients ∂. This coefficient then adaptively fuses the two branch features through a gated weighted summation fusion strategy, ultimately yielding the output feature Out. The network structures of the MSAGBlock, CAMBlock and MCB are illustrated in Fig 3c, 3d and 3e, respectively.


$$\begin{array}{c} {\;D}_{CSU}\;=\;RELU(CS(BN({DSConv}_{\text{9}\times \text{9}}(UP(d;\;\text{2}))))) \end{array}$$ (5)

$$\begin{array}{c} Out\;=\;\partial {\;⨀\;Y}_{MSAG}(g,\;x)\;+\;(\text{1}-\partial )\;⨀\;Y_{CAM}(x) \end{array}$$ (6)

#### 3.3.1. Multi-scale spatial attention gated block (MSAGBlock).

MSAGBlock achieves selective amplification or suppression of local spatial details while capturing global information through two multi-scale convolutional blocks (MCB), a dual gating (DG) mechanism with Sigmoid and Tanh activations, and a gated feature transformation (GFT) operation. The following details the implementation process of MSAGBlock:

First, we introduce the global feature g ∈ R^Cg×H×W^ and the local feature x ∈ R^Cx×H×W^ to the MCB (MCB(∙)), yielding f_g_ and f_x_ ∈ R^Cint×H×W^ (where C_int_ denotes the number of intermediate channels).


$$\begin{array}{c} f_{g}\;=\;MCB(g) \end{array}$$ (7)

$$\begin{array}{c} f_{x}\;=\;MCB(x)\; \end{array}$$ (8)

Then, the aggregated results from both blocks are summed, followed by a 1 × 1 convolution, BN, and GELU activation to fuse features containing multi-scale context, generating F ∈ R^Cint×H×W^:


$$\begin{array}{c} F\;=\;GELU(BN({Conv}_{\text{1}\times \text{1}}(f_{g}\;+\;f_{x}))) \end{array}$$ (9)

Next, F is introduced into the DG. The DG comprises two parallel gated branches: one generates the gated feature map A via a 1 × 1 convolution with Sigmoid activation, controlling feature retention and suppression; the other produces the gated feature map M through a 1 × 1 convolution with Tanh activation, enabling fine-grained modulation in both positive and negative directions on the features.


$$\begin{array}{c} A\;=\;Sigmoid({Conv}_{\text{1}\times \text{1}}(F)){\;\in \;\left(\text{0},\;\text{1}\right)}^{C_{int}\times H\times W} \end{array}$$ (10)

$$\begin{array}{c} M\;=\;Tanh({Conv}_{\text{1}\times \text{1}}(F))\;\in \;{\left(-\text{1},\;\text{1}\right)}^{C_{int}\times H\times W} \end{array}$$ (11)

Subsequently, the local feature x is projected to an intermediate channel dimension C_int_ through a 1 × 1 convolution and BN, producing the output x_lat_ ∈ R^Cint×H×W^. Then, A and M respectively perform element-wise multiplication with the x_lat_ feature to conduct the GFT operation. After summation, the preliminary output feature Y is obtained.


$$\begin{array}{c} Y\;={\;x}_{lat}\;⨀\;A\;+{\;x}_{lat}\;⨀\;M \end{array}$$ (12)

Finally, Y is mapped back to the number of channels consistent with x via a pointwise convolution, then apply BN to get the final output feature Y_MSAG_∈R^Cx×H×W^.

#### 3.3.2. Channel attention modulation block (CAMBlock).

Inspired by the design principles of ConvNeXt [37], we propose an improved and more efficient CAMBlock. In this block, we use a 3 × 3 depthwise convolution (DWConv_3 × 3_(·)) and group normalization (GN(‧)) to better capture details of small targets. The channel MLP applies a nonlinear transformation to the channel vector at each spatial position using two 1 × 1 convolutions combined with a GELU activation, thus enhancing the expression of information between channels. On this basis, we incorporate the SE module [23] to adaptively weight the output of the channel MLP, evaluating the importance of each channel. Additionally, LayerScale, DropPath, and residual connection are integrated to maintain gradient stability and support convergence in deep networks. The overall computational process can be expressed as follows:


$$\begin{array}{c} Y_{CAM}\;=\;x\;+\;DropPath(\gamma \;⨀\;SE(\overset{\text{1}\times \text{1}}{\underset{\text{2}}{Conv}}(GELU(\overset{\text{1}\times \text{1}}{\underset{\text{1}}{Conv}}(GN({DWConv}_{\text{3}\times \text{3}}(x))))))) \end{array}$$ (13)

Here, Conv_1_ is used to expand the number of channels: R^C^ → R^4C^; Conv_2_ is used to compress back to the original number of channels: R^4C^→R^C^. γ represents LayerScale.

## 4. Experiments

### 4.1. Datasets

1) Synapse [38]: This dataset originates from the MICCAI 2015 Multi-Organ Abdominal Labeling Challenge. It comprises 30 abdominal CT cases totaling 3779 axial CT slices. Each slice is annotated for eight organ classes, including the liver, stomach, left kidney, right kidney, aorta, pancreas, gallbladder, and spleen. In our study, we follow the commonly used split of 18 cases for training and 12 cases for testing.
2) ACDC [39]: This dataset contains short-axis cardiac image sequences from 100 patients, providing detailed annotations for the left ventricle, myocardium, and right ventricle by professional clinicians. We partitioned it into a training set (70%), a validation set (20%), and a test set (10%).

### 4.2. Evaluation indicators

**Dice similarity coefficient (DSC):** The similarity between the predicted segmentation and the ground truth is evaluated based on their overlapping regions. The value ranges from 0 to 1, with values closer to 1 indicating that the predicted result matches the true labeling more closely.


$$\begin{array}{c} DSC\;=\;\frac{\text{2}\;\times \;\left|M_{p\;}\cap {\;M}_{g}\right|}{\left|M_{p}\right|\;+\;\left|M_{g}\right|} \end{array}$$ (14)

Here, M_p_ represents the predicted segmentation region, and M_g_ corresponds to the ground truth annotation region. |M_p_| and |M_g_| represent the total number of pixels within their respective regions.

**95% Hausdorff distance (HD95):** The impact of outliers is reduced by calculating the 95th percentile of all boundary point distances (i.e., excluding the most extreme 5% of distance values). A lower HD95 indicates higher consistency between the predicted boundaries and the actual boundaries.


$$\begin{array}{c} HD\text{95 }=\;max(d_{\text{95}}(R_{\text{1}},\;R_{\text{2}}),{\;d}_{\text{95}}(R_{\text{2}},{\;R}_{\text{1}})) \end{array}$$ (15)

Here, R_1_ and R_2_ denote the sets of boundary points for the predicted region and the actual annotated region, respectively. d_95_(R_1_, R_2_) denotes the 95th percentile of the distance from each point in R_1_ to the nearest point in R_2_.

### 4.3. Specific details

Our development environment includes Python 3.8, PyTorch 1.11.0, Ubuntu 20.04, and CUDA 11.3, with an NVIDIA RTX 3090 (24GB) as the computing device. To ensure reproducibility, all experiments were conducted using a fixed random seed of 1234. Training parameters were set according to the datasets. For the Synapse dataset, we trained for 30 epochs with an initial learning rate of 1e^-3^ and a minimum learning rate of 0. For the ACDC dataset, we trained for 150 epochs with an initial learning rate of 5e^-4^ and a minimum learning rate of 0. All experiments used an input size of 224 × 224 and a fixed batch size of 24. We employed the AdamW optimizer along with a cosine annealing learning rate scheduler. A hybrid loss function, consisting of a weighted combination of cross-entropy loss and dice loss, was used, with the weighting coefficients α and β adjusted according to the dataset. Specifically, for Synapse, both coefficients were set to 0.5, while for ACDC, they were set to 0.3 and 0.7, respectively. To accelerate training convergence, we initialized the encoder with pre-trained weights from RWKV-UNet Enc-B [33].

During resampling, the original images in the Synapse and ACDC datasets were processed using bicubic interpolation and nearest-neighbor interpolation, respectively, while all segmentation labels uniformly employed nearest-neighbor interpolation. Subsequently, all two-dimensional image slices across both datasets were uniformly scaled to a fixed resolution of 224 × 224 to ensure consistent input dimensions for the network. During training, we performed online random data augmentation operations: each sample underwent geometric transformations with a 50% probability, consisting of rotations in multiples of 90° (0°, 90°, 180°, 270°) combined with random horizontal or vertical flips. If this augmentation was not triggered, a random planar rotation within the range of −20° to +20° was further applied with a 25% probability. No augmentation was applied in other cases. During the testing phase, deterministic test-time augmentation is introduced to enhance prediction robustness. Fixed transformations vary by dataset: the Synapse dataset uses only horizontal flipping, while the ACDC dataset employs horizontal flipping alongside rotations at 0°, 90°, 180°, and 270°. Predictions from the same input under different deterministic transformations are ultimately fused to produce the final segmentation result.

## 5. Analysis

### 5.1. Comparison with other methods

The methods we compared include U-Net [8], TransUNet [15], ViT + CPU [15], Swin-Unet [16], Attention U-Net [28], LeViT-UNet-384 [17], MT-UNet [18], SA-UMamba [25], RWKV-UNet [33], PVT-CASCADE [40], TransCASCADE [40], MS-UNet [41], TransDeepLab [42], VM-UNet [43], STA-UNet [44], SelfReg-UNet [45], SelfReg-SwinUNet [45], and MISSFormer [46]. Among them, RWKV-UNet served as the direct baseline model for this study, and its results were re-implemented by us in the development environment of this paper and labeled with “re-impl” in the tables. To validate the performance gains of our proposed model over RWKV-UNet under fair comparison conditions, our method employs identical training strategies, hyperparameters, and other configurations to RWKV-UNet. For all other comparison methods, we did not retrain models or perform additional hyperparameter tuning. Instead, we directly adopted the optimal results reported in their respective original papers on the same datasets to avoid introducing potential unfair comparisons due to training details or inconsistent tuning strategies. It is worth noting that after this study was completed, the authors of RWKV-UNet released improved results based on parameter adjustments and data augmentation strategies. However, to maintain consistency and fairness in comparisons, all experimental results for our method and the RWKV-UNet re-implementation are based on its original publicly available version (released January 14, 2025), rather than subsequent improved version.

In the Synapse multi-organ segmentation task, SCEAF-UNet significantly outperforms the previous SOTA methods on both the average DSC and average HD95 metrics, while also surpassing the baseline model RWKV-UNet (re-impl), as shown in Table 1. Based on CNN models (U-Net [8], Attention U-Net [28]), SCEAF-UNet achieves average DSC improvements of 8.02% and 7.1%, while reducing the average HD95 by 25.59 mm and 21.91 mm, respectively. Compared to Transformer-based models (Swin-Unet [16], TransDeepLab [42], MT-UNet [18]), it achieves average DSC improvements of 5.74%, 4.71%, and 6.28%, while reducing average HD95 by 7.44 mm, 7.14 mm, and 12.48 mm. Relative to the baseline model RWKV-UNet (re-impl), our approach further elevates the average DSC to 84.87% and reduces the average HD95 to 14.11 mm, compensating for the shortcomings of the RWKV-UNet (re-impl) in segmenting organs such as the gallbladder and spleen. Overall, SCEAF-UNet maintains the strengths of the baseline model while achieving steady improvements in global performance and exhibits greater robustness.

**Table 1:** A comparative analysis of multiple methods on the Synapse dataset. Note: Ao: aorta, Ga: gallbladder, LKi: left kidney, RKi: right kidney, Li: liver, Pa: pancreas, Sp: spleen, St: stomach. Here, -- denotes missing data, ↑↓ indicates higher (lower) is better; Bold-type and underlined entries indicate the best and second-ranked performances, respectively; DSC is measured in %, HD95 in mm (as below).

Methods	Avg DSC↑	Avg HD95↓	Ao	Ga	LKi	RKi	Li	Pa	Sp	St
TransUNet [15]	77.48	31.69	87.23	63.16	81.87	77.02	94.08	55.86	85.08	75.62
Attention U-Net [28]	77.77	36.02	89.55	68.88	77.98	71.11	93.57	58.04	87.30	75.75
TransDeepLab [42]	80.16	21.25	86.04	69.16	84.08	79.88	93.53	61.19	89.00	78.40
U-Net [8]	76.85	39.70	89.07	69.72	77.77	68.60	93.43	53.98	86.67	75.58
MS-UNet [41]	80.44	18.97	85.80	69.40	85.86	81.66	94.24	57.66	90.53	78.33
VM-UNet [43]	81.08	19.21	86.40	69.41	86.16	82.76	94.17	58.80	89.51	81.40
STA-UNet [44]	80.69	--	89.10	68.34	84.97	79.44	93.39	63.32	88.69	78.26
Swin-Unet [16]	79.13	21.55	85.47	66.53	83.28	79.61	94.29	56.58	90.66	76.60
MISSFormer [46]	81.96	18.20	86.99	68.65	85.21	82.00	94.41	65.67	91.92	80.81
SelfReg-UNet [45]	80.34	--	88.74	71.78	85.32	80.71	93.80	62.22	84.78	75.39
SelfReg-SwinUNet [45]	80.54	--	86.07	69.65	85.12	82.58	94.18	61.08	87.42	78.22
MT-UNet [18]	78.59	26.59	87.92	64.99	81.47	77.29	93.06	59.46	87.75	76.81
SA-UMamba [25]	82.54	16.80	88.07	70.46	86.46	83.96	94.42	65.32	89.89	81.76
RWKV-UNet [33]	84.02	15.70	89.53	68.94	87.63	**84.07**	**95.57**	69.38	90.95	86.09
RWKV-UNet (re-impl)	83.03	20.23	89.20	66.50	87.20	81.42	95.53	68.67	90.45	85.28
SCEAF-UNet (ours)	**84.87**	**14.11**	**89.73**	**72.10**	**88.12**	83.91	95.46	**71.22**	**92.17**	**86.24**

In the ACDC dataset, our method still maintains an advantage. As shown in Table 2, compared to U-Net [8] and Swin-Unet [16], SCEAF-UNet improves the average DSC by 2.64% and 2.32%, respectively. Relative to the RWKV-UNet (re-impl), our method achieves a 0.74 percentage point improvement in average DSC. At the organ level, compared to TransCASCADE [40], our method still lags behind in segmenting myocardial tissue.

**Table 2:** A comparative study of various approaches on the ACDC dataset.

Methods	Avg DSC↑	Right ventricle	Myocardium	Left ventricle
U-Net [8]	89.68	87.17	87.21	94.68
LeViT-UNet-384 [17]	90.32	89.55	87.64	93.76
Swin-Unet [16]	90.00	88.55	85.62	95.83
TransCASCADE [40]	91.63	89.14	**90.25**	95.50
MISSFormer [46]	90.86	89.55	87.64	93.76
ViT + CPU [15]	81.45	81.46	70.71	92.18
PVT-CASCADE [40]	91.46	88.90	89.97	95.50
MS-UNet [41]	87.74	85.31	84.09	93.82
TransUNet [15]	89.71	88.86	84.53	95.73
RWKV-UNet [33]	92.17	90.86	88.72	96.92
RWKV-UNet (re-impl)	91.58	90.16	88.26	96.32
SCEAF-UNet (ours)	**92.32**	**91.39**	88.59	**96.97**

### 5.2. Computational efficiency analysis

To comprehensively evaluate the computational efficiency of the proposed method in real-world deployment scenarios, we further conducted a systematic analysis of the model’s inference time and GPU memory consumption. Due to differences in network implementation, inference frameworks, and optimization strategies among various comparison methods, this paper focuses solely on reporting the actual inference efficiency of the proposed method and the direct baseline model under standardized settings to ensure reproducibility and interpretability of results. All experiments were conducted under identical hardware conditions using a batch size of 1 to simulate the practical application scenario of processing individual medical images on a case-by-case basis in clinical reasoning. As shown in Table 3, SCEAF-UNet exhibits only a slight increase in parameters and memory consumption compared to the baseline model. Accordingly, its average inference time per image is 20.59 ms, increasing by only approximately 1.8 ms compared to RWKV-UNet’s 18.76 ms. Both methods exhibit nearly identical peak GPU memory usage during inference, indicating that the introduced structural improvements do not impose additional memory overhead.

**Table 3:** Comparative experiments on inference time and memory consumption between SCEAF-UNet and RWKV-UNet.

Method	Params(M)	FLOPs(G)	Avg time(ms)	GPU memory(M)
RWKV-UNet	17.37	11.11	18.76 ± 1.81	1556.0
SCEAF-UNet	17.52	11.40	20.59 ± 1.28	1556.6

Overall, SCEAF-UNet demonstrates a favorable trade-off between computational efficiency and performance, showing potential to meet the demands of near-real-time clinical applications on modern GPU platforms.

### 5.3. Ablation and analysis

In order to corroborate the rationality and practicality of the designed SCEAF-UNet architecture, we conducted the following ablation experiments on the Synapse dataset.

The proposed MSAGblock, CAMBlock, and EAF module enhance segmentation accuracy at spatial, channel, and boundary levels. Table 4 presents ablation results demonstrating the effectiveness of each module, revealing independent contributions to segmentation performance. The EAF module enhances encoded features through edge attention, accentuating target boundaries. This boosts the average DSC from 80.40% to 82.48% and reduces the average HD95 from 27.80 mm to 23.20 mm. It introduces only approximately 0.33 M parameters and 0.72 GFLOPs, demonstrating a high performance-computational efficiency ratio. Adding either the CAMBlock or MSAGBlock on top of the EAF module incurs minimal computational overhead (CAMBlock adds 0.06 M parameters and 0.13 GFLOPs; MSAGBlock adds 0.04 M parameters and 0.07 GFLOPs) while delivering significant improvements in boundary accuracy. When all three modules are combined, the complete SCEAF-UNet requires only approximately 0.45 M additional parameters and 1.5 GFLOPs compared to the base model without any modules. This achieves a 4.47% average DSC improvement and a 13.71 mm reduction in average HD95, fully demonstrating the proposed modules’ excellent balance between performance gains and computational overhead. It also validates the complementary relationship between the EAF module, MSAGblock, and CAMBlock in boundary refinement, spatial detail restoration, and channel feature discrimination.

**Table 4:** Ablation experiments for each module in the SCEAF-UNet. Here, MSAG, CAM, and EAF represent the MSAGBlock, CAMBlock, and EAF module, respectively.

MSAG	CAM	EAF	Params(M)	FLOPs(G)	Avg DSC↑	Avg HD95↓
No	No	No	17.07	9.90	80.40	27.82
No	No	Yes	17.40	10.62	82.48	23.20
No	Yes	Yes	17.46	10.75	82.95	18.41
Yes	No	Yes	17.44	10.69	83.15	17.47
Yes	Yes	Yes	17.52	11.40	84.87	14.11

The choice of feature fusion strategy has a notable impact on segmentation accuracy [47,48]. To investigate this, we conducted ablation experiments on five different fusion approaches: cascade, addition, concatenation, multiplication, and gated weighted summation. The results are summarized in Table 5. It can be seen that although cascade, multiplication, and addition introduced no extra computational costs compared to the gated weighted summation, their segmentation performance remained relatively limited. Concatenation incurred the highest training cost while yielding the lowest performance. By contrast, the gated weighted summation strategy adopted in our study achieves the best results, with an average DSC of 84.87% and an average HD95 of 14.11 mm. This indicates that this strategy effectively balances spatial and channel feature modeling, enhancing both feature representations and fusion efficiency.

**Table 5:** Ablation experiments for MSAGBAlock and CAMBlock fusion strategies.

Fusion Method	Params(M)	FLOPs(G)	Avg DSC↑	Avg HD95↓
Cascade	17.52	11.40	83.97	18.22
Addition	17.52	11.40	83.58	18.59
Concatenation	17.54	11.49	82.02	21.12
Multiplication	17.52	11.40	83.71	15.64
Gated weighted summation	17.52	11.40	84.87	14.11

After processing the multi-scale features obtained during the encoding stage through the EAF module, the model’s contour perception capability is effectively enhanced, achieving outstanding performance on both DSC and HD95 metrics. In this ablation study, we set the number of EAF modules to 0, 1, 2, and 3 to analyze the impact on segmentation results by introducing different quantities of EAF modules into the skip connections. Notably, while EAF modules are introduced to improve HD95 values, this paper primarily uses DSC as the core evaluation metric. Therefore, we focus on analyzing the improvement in DSC.

**Fig 4:**
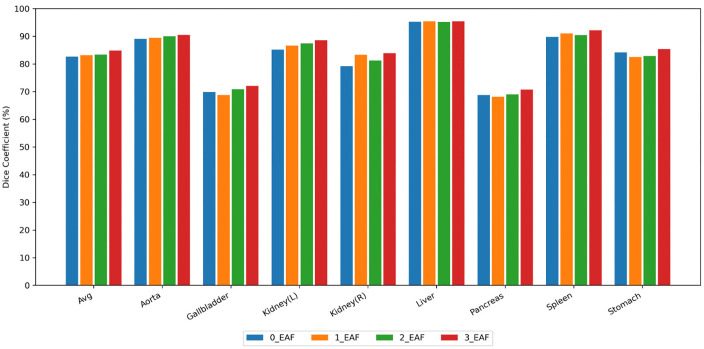
Segmentation results with varying numbers of EAF modules integrated into the skip connections.

As shown in Fig 4, the overall average DSC of the model exhibits a continuous upward trend with increasing numbers of EAF modules. When introducing one or two EAF modules, the performance improvement across organs is relatively limited, and DSC values for some organs even show slight decreases. When the number increases to three, the segmentation accuracy improves across all organs to varying degrees, yielding the best overall results. All in all, as EAF module is progressively increased to the skip connection, its role in enhancing boundary features becomes increasingly prominent.

We went on to explore the performance of the SCEAF module at different locations in the decoder network and the results obtained are shown in Table 6. We find that the model performs best when the module is placed in the second and third stages of the decoder. In contrast, integrating the module solely into the D_1_ stage generates the poorest average DSC value. This may stem from the fact that feature resolution at this stage has largely recovered to the input scale, where insufficient semantic information prevents the SCEAF module from modeling effectively.

**Table 6:** Results of embedding the SCEAF module at different decoding stages. Here, D_4_ to D_1_ represent the deepest to shallowest stages of the decoder, respectively.

Decoder layer	Params(M)	FLOPs(G)	Avg DSC↑	Avg HD95↓
D_4_	17.71	10.86	82.04	21.70
D_1_	17.41	11.25	81.52	23.72
D_4_ + D_3_	17.80	11.13	84.35	15.39
D_2_ + D_1_	17.45	11.76	83.56	20.20
D_4_ + D_3_ + D_2_	17.84	11.64	82.17	24.26
D_4_ + D_3_ + D_2_ + D_1_	17.85	12.27	83.86	18.03
D_3_ + D_2_	17.52	11.40	84.87	14.11

To validate the efficacy of each key component in the designed MSAGBlock, we performed stepwise ablation tests on MCB, DG, and GFT, as shown in Table 7. The findings indicate that introducing MCB obviously improves the average DSC while reducing the average HD95, proving that our designed MCB can effectively capture spatial details at different scales and offers significant advantages in multi-scale feature representations. GFT and DG, as core components of the module, both enhance segmentation accuracy when used in conjunction with MCB. As we enabled all three components, the model achieves the best results on both metrics, verifying the rationality and effectiveness of the MSAGBlock design.

**Table 7:** Ablation study for key components of the MSAGBlock.

MCB	DG	GFT	Params(M)	FLOPs(G)	Avg DSC↑	Avg HD95↓
No	No	No	17.47	11.08	83.08	20.89
Yes	No	Yes	17.52	11.37	83.99	13.09
No	Yes	Yes	17.48	11.13	83.34	22.54
Yes	Yes	No	17.52	11.37	83.46	16.58
Yes	Yes	Yes	17.52	11.40	84.87	14.11

CAMBlock is our proposal inspired by the design philosophy of ConvNeXt [37]. Our primary improvements involve two key modifications: replacing ConvNeXt’s 7 × 7 depthwise convolution with a 3 × 3 convolution, and introducing the SE module [23]. As indicated by the results in Table 8, when the CAMBlock adopts a large 7 × 7 convolution kernel, its average DSC decreases by 0.17% and the average HD95 increases by 3.26 mm compared with the original ConvNeXt module. However, as the kernel size decreases to 5 × 5, overall segmentation performance begins to improve, reaching its peak when adjusted to 3 × 3. These results demonstrate that reducing the size of the convolutional kernel while introducing the SE module is both highly effective and the correct approach.

**Table 8:** Ablation results comparing CAMBlock and ConvNeXt.

Method	Kernel size	Params(M)	FLOPs(G)	Avg DSC↑	Avg HD95↓
ConvNeXt	7	17.53	11.43	83.00	20.44
	5	17.52	11.41	82.95	21.86
	3	17.52	11.40	83.32	13.81
CAMBlock	7	17.53	11.43	82.83	23.70
	5	17.53	11.41	83.23	16.04
	3	17.52	11.40	84.87	14.11

To analyze the sensitivity of key hyperparameters, we further investigated the impact of the channel reduction factor r in the EAF module and the intermediate channel dimension C_int_ in the MSAGBlock on model performance and computational overhead.

For the channel reduction factor r in the EAF module, we conducted ablation experiments by setting it to 2, 4, 8, and 16 respectively. The results in Table 9 indicate that when r = 4 (the configuration used in this paper), the model achieves the optimal balance between segmentation accuracy and computational complexity. A smaller r increases the computational cost, while a larger r may lead to a decrease in the ability to model fine-grained edge information.

**Table 9:** Ablation test results for channel reduction factor r in the EAF module.

Reduction ratio(r)	Params(M)	FLOPs(G)	Avg DSC↑	Avg HD95↓
r = 2	17.54	11.43	83.59	14.13
r = 8	17.52	11.38	83.37	15.32
r = 16	17.51	11.37	83.23	18.38
r = 4	17.52	11.40	84.87	14.11

We conducted ablation experiments with different C_int_ settings while keeping the network structure, training strategy and other hyperparameters identical. Specifically, C_int_ was set to the number of input feature channels C, C/2 and C/4. The experimental results are shown in Table 10. It can be observed that when no compression is applied to the channels (C_int_ = C), the number of model parameters and the computation amount are high, but the segmentation performance is not satisfactory, indicating that too large intermediate channel dimensions do not lead to effective performance improvement. On the contrary, when C_int_ is too small (C_int_ = C/4), the model capacity is limited, leading to a decrease in segmentation accuracy. In contrast, the model achieves the best balance between segmentation accuracy and computational efficiency when a medium channel compression ratio (C_int_ = C/2) is used.

**Table 10:** Ablation study results for intermediate channel dimension C_int_ in MSAGBlock. where C denotes the channel dimension of the corresponding stage feature.

C_int_	Params(M)	FLOPs(G)	Avg DSC↑	Avg HD95↓
C	17.58	11.73	83.88	19.73
C/4	17.50	11.25	83.12	19.35
C/2	17.52	11.40	84.87	14.11

### 5.4. Statistical analysis

In order to verify that the performance improvement of SCEAF-UNet over the baseline method RWKV-UNet is statistically significant, the experimental results of the test set are systematically analyzed in this paper. Considering the small test sample size (12 samples) of the Synapse dataset and the fact that the segmentation metrics may not satisfy the assumption of normal distribution, this paper employed nonparametric statistical methods.

Specifically, for the segmentation results of each test sample, the paired Wilcoxon signed-rank test was employed to perform statistical significance analysis on the performance differences between SCEAF-UNet and the baseline method regarding the DSC and HD95 metrics, with a significance level set at α = 0.05. The results in Table 11 indicate that SCEAF-UNet achieved a statistically significant improvement over the baseline method in the DSC metric (p = 0.0093), while no statistical significance was reached in the HD95 metric (p = 0.3804). Nevertheless, its average HD95 value remained lower than that of the baseline method, suggesting the model demonstrates certain improvements in boundary localization accuracy.

**Table 11:** Results of statistical significance analysis using the paired Wilcoxon signed-rank test for baseline and SCEAF-UNet (Mean Δ = SCEAF-UNet − baseline).

Metric	P-value	Significance	Mean ∆	95% CI
DSC	0.0093	Significant	+0.0184	[+0.0072, +0.0315]
HD95	0.3804	Not significant	−6.12	[−13.17, +0.08]

Furthermore, to assess the stability and uncertainty of model performance, this study employed bootstrap resampling (1000 iterations) to estimate 95% confidence intervals for both DSC and HD95 metrics. Results demonstrate that the performance improvement in DSC remains consistent across different resampling conditions, further validating the stability of the model enhancement. While HD95 exhibits some fluctuation in individual samples, it overall maintains an improving trend.

### 5.5. Visualization experiments and analysis

**Fig 5:**
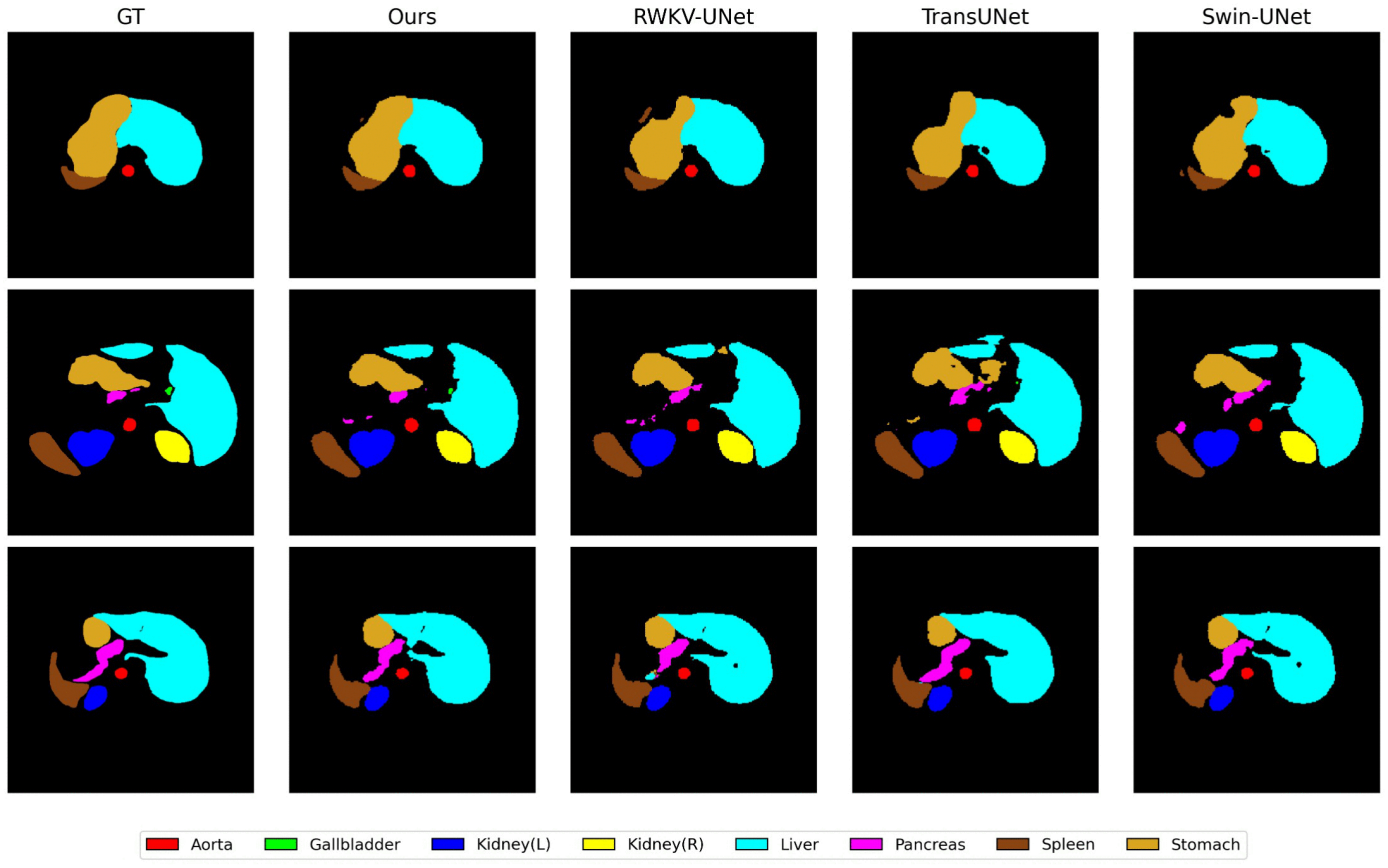
Comparison of the visualized segmentation results among different methods.

As shown in Fig 5, we conducted a qualitative comparison of segmentation results from different methods on the Synapse dataset [38], where different colors correspond to different organ regions.

We observe that compared to RWKV-UNet, TransUNet, and Swin-UNet, SCEAF-UNet generates more continuous and anatomically accurate segmentation results on elongated and irregularly shaped organs (pancreas, gallbladder). This improvement is primarily attributed to the SCEAF module introduced in the decoding stage. Through the adaptive fusion of multi-scale large kernel spatial modeling and channel interaction modeling, this module is able to effectively balance the global structure perception and local discriminative ability in the neighboring regions of complex organs, thus alleviating the problem that fine organs are easily damaged during the up-sampling process. Similarly, in large organ regions like the liver and stomach, its predictions exhibit higher consistency with ground truth in overall morphology and boundary positioning. This is mainly due to the significant contribution of the introduced EAF module. By explicitly modeling high-frequency edge responses, this module guides the network to focus more on true anatomical boundaries, significantly improving contour localization accuracy. The combination of these two modules enables the model to achieve finer and more reliable organ segmentation results while maintaining semantic consistency. This phenomenon aligns with the trends observed in quantitative experiments, where Dice scores increase and HD95 values decrease.

### 5.6. Analysis of failure cases

**Fig 6:**
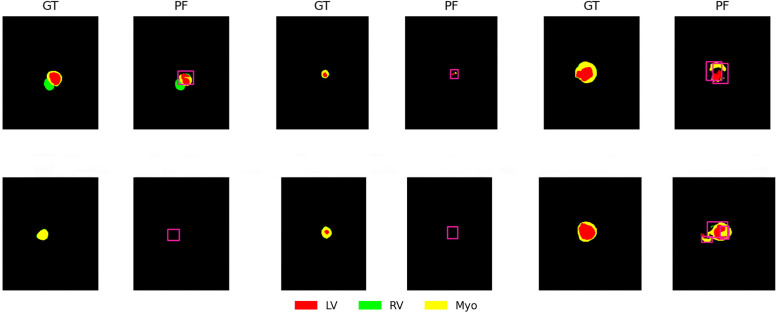
Our method encountered some failures on myocardial segments within the ACDC dataset. Here, GT denotes ground truth, and PF denotes prediction failure.

Although the method in this paper achieves competitive overall performance on the ACDC dataset [39], its performance on myocardial segmentation is relatively poor. To further analyze this limitation, the segmentation error is systematically analyzed in this paper and representative failure cases are given. As shown in Fig 6, we are able to see significant under-segmentation of the myocardial region predicted by the model, especially in the apical slices and the low-contrast cardiac cycle phase. This may be attributed to the fact that compared with the left ventricle and right ventricle, the myocardium is characterized by a thin structure, low boundary contrast, and significant deformation across slices, making it more sensitive to spatial localization errors. The left ventricle is accurately segmented in most slices, but significant discrepancies occur in some low-contrast slices and those near the apex or base of the heart. This phenomenon is typically caused by the propagation of myocardial segmentation errors.

To address this challenge, we will pursue improvements from multiple angles in subsequent work. First, incorporating boundary-aware or shape-constrained loss functions will enhance the integrity of myocardial contours. Second, integrating higher-resolution multi-scale features during decoding effectively preserves fine-grained spatial information. Additionally, designing targeted data augmentation or curriculum learning strategies for apical slices and low-contrast samples may mitigate the impact of data scarcity. These enhancements are expected to further improve myocardial segmentation performance without significantly increasing model complexity.

## 6. Discussion

The proposed method was validated on two representative medical imaging datasets: abdominal CT (Synapse) and cardiac MRI (ACDC). However, further experimentation and analysis are needed regarding its generalization capabilities across other imaging modalities and anatomical regions, as well as the design choices that may limit its generalization.

### 6.1. Applicability to other imaging modalities

From the perspective of model architecture, the proposed method does not rely on artificially designed features for CT or MRI, making it theoretically extensible to other imaging modalities such as ultrasound, X-ray, or mammography. However, these modalities exhibit differences from CT and MRI in terms of noise types, imaging contrast mechanisms, and artifact characteristics. Examples include speckle noise in ultrasound and structural overlap issues arising from X-ray projection imaging. In specific experiments, model performance may be compromised without incorporating corresponding modality-adaptive preprocessing or normalization strategies.

### 6.2. Generalizability to other anatomical regions

Beyond the abdomen and heart, this method holds promise for extension to anatomical regions with similar spatial continuity and organ scales, such as pelvic or thoracic structures. However, segmentation difficulty increases significantly in regions dominated by slender, thin-walled, or highly deformable structures (e.g., vessels or small lesions), consistent with the error patterns observed in myocardial segmentation in this paper. In such scenarios, targeted structural constraints or feature enhancement mechanisms may be required.

### 6.3. Design choices that may limit generalization

First, fixed input resolutions may limit the model’s ability to capture fine-scale features across different spatial scales. Second, current training strategies primarily rely on 2D slices, failing to fully leverage the 3D contextual information inherent in volumetric data. Finally, despite employing deterministic and fair test-time augmentation strategies, their configurations remain dataset-dependent and may not be universally applicable across all imaging modalities.

## 7. Conclusions

In this study, we build an advanced network architecture, SCEAF-UNet. It incorporates the proposed SCEAF module to improve spatial detail recovery and channel feature representations. Moreover, the integrated EAF module further enhances boundary perception capabilities, allowing for more precise segmentation of complex organ structures. Extensive experimental results show that SCEAF-UNet outperforms RWKV-UNet [33] as well as other SOTA models, highlighting great potential for practical application in clinical settings.
